# Analysis of Intestinal Mycobiota of Patients with Clostridioides difficile Infection among a Prospective Inpatient Cohort

**DOI:** 10.1128/spectrum.01362-22

**Published:** 2022-07-14

**Authors:** Yangchun Cao, Lamei Wang, Shanlin Ke, Ciarán P. Kelly, Nira R. Pollock, Javier A. Villafuerte Gálvez, Kaitlyn Daugherty, Hua Xu, Junhu Yao, Yulin Chen, Yang-Yu Liu, Xinhua Chen

**Affiliations:** a College of Animal Science and Technology, Northwest A&F University, Yangling, Shaanxi Province, China; b Division of Gastroenterology, Department of Medicine, Beth Israel Deaconess Medical Center, Harvard Medical School, Boston, Massachusetts, USA; c Channing Division of Network Medicine, Department of Medicine, Brigham and Women’s Hospital and Harvard Medical School, Boston, Massachusetts, USA; d Division of Infectious Diseases, Department of Medicine, Beth Israel Deaconess Medical Center, Harvard Medical School, Boston, Massachusetts, USA; e Department of Laboratory Medicine, Boston Children’s Hospital, Harvard Medical School, Boston, Massachusetts, USA; Ohio State University

**Keywords:** *Clostridiodes difficile*, ITS2, biomarker, diarrhea, gut mycobiota

## Abstract

Clostridioides difficile infection (CDI) is a burden to health care systems worldwide. Gut microbiota dysbiosis associated with CDI has been well accepted. However, contribution of fungal mycobiota to CDI has recently gained research interest. Here, we report the gut mycobiota composition of 149 uniquely well characterized participants from a prospective clinical cohort and evaluate the discriminating ability of gut mycobiota to classify CDI and non-CDI patients. Fecal samples were divided into two groups: (i) CDI (inpatients who had clinically significant diarrhea and positive nucleic acid amplification testing [NAAT] and received subsequent CDI therapy, *n* = 58) and (ii) non-CDI, which can be further divided into three subgroups: (a) carrier (inpatients with positive stool NAAT but without diarrhea; *n* = 28); (b) diarrhea (inpatients with negative stool NAAT; *n* = 31); and (c) control (inpatients with negative stool NAAT and without diarrhea; *n* = 32). Fecal mycobiota composition was analyzed by internal transcribed spacer 2 (ITS2) sequencing. In comparison to non-CDI patients, CDI patients tend to have gut mycobiota with lower biodiversity, weaker fungi correlations, and weaker correlations between fungi and host immune factors. Notably, 11 genera (Saccharomyces, Penicillium, Aspergillus, Cystobasidium, Cladosporium, and so on) were significantly enriched in non-CDI patients, and Pichia and Suhomyces were enriched in patients with CDI, while 1 two genera, Cystobasidium and Exophiala, had higher abundance in patients with diarrhea compared with CDI (linear discriminant analysis [LDA] > 3.0; *P < *0.05). Ascomycota and Basidiomycota (or Candida and Saccharomyces) exhibited a strong negative correlation (*r* ≤ −0.714 or *r* ≤ −0.387; *P < *0.05), and the ratios of Ascomycota to Basidiomycota or genera Candida to Saccharomyces were dramatically higher in CDI patients than in non-CDI patients (*P < *0.05). A disease-specific pattern with much weaker fungal abundance correlations was observed in the CDI group compared to that in the non-CDI and diarrhea groups, suggesting that these correlations may contribute to the development of CDI. Our findings provided specific markers of stool fungi that distinguish CDI from all non-CDI hospitalized patients. This study’s potential clinical utility for better CDI diagnosis warrants further investigation.

**IMPORTANCE** Clostridioides difficile is an opportunistic bacterial pathogen that causes a serious and potentially life-threatening infection of the human gut. It remains an existing challenge to distinguish active infection of CDI from diarrhea with non-CDI causes. A few large prospective studies from recent years suggest that there is no single optimal test for the diagnosis of CDI. Previous research has concentrated on the relationship between bacteria and CDI, while the roles of fungi, as a significant proportion of the gut microbial ecosystem, remain understudied. In this study, we report a series of fungal markers that may add diagnostic values for the development of a more systematic approach to accurate CDI diagnosis. These results help open the door for better understanding of the relationship between host immune factors and the fungal community in the context of CDI pathogenesis.

## INTRODUCTION

Clostridiodes difficile (formerly Clostridium difficile) is the leading cause of health care-associated infections. Each year in the United States, over half million cases of C. difficile infection (CDI) are associated with over 29,000 associated deaths, with attributable costs of over $5.4 billion (1–3). About 1 to 3% of hospitalized patients become infected with C. difficile, and many experience recurrent CDI. The high relapse rates of CDI may be partially due to the disruption of the gut microbiota (4).

Current CDI diagnosis methods include detecting toxinogenic C. difficile (nucleic acid amplification testing [NAAT] or culture), the C. difficile toxins, and algorithmic test combinations (5). We previously found that neither stool toxin concentration nor NAAT cycle threshold value can accurately distinguish a CDI patient from a colonized patient with diarrhea from another cause (5, 6). Previous studies have demonstrated that inflammation markers (including cytokines, calprotectin, fecal lactoferrin, and calprotectin) are not specific to CDI and thus could not be sufficient for diagnosis as biomarker (7). The identification of a biomarker to differentiate CDI from non-CDI inpatients will be helpful to improve CDI diagnosis.

Both genetic and environmental factors play important roles in CDI pathogenesis, and environmental factors are more important than genetic predisposition (8, 9). Recent studies revealed that disruption of the gut microbiome (e.g., dysbiosis) could increase the risk of CDI by disrupting the gut microbiome’s ability to resist pathogen colonization or by weakening the intestinal barrier, thereby promoting infection (2, 4, 10–12). Our previous study revealed that CDI is associated with alteration of many different aspects of the gut microbiota, including overall microbial diversity and microbial association networks (13). We also provided evidence that gut microbiota and host immune markers can be used for distinguishing CDI from carrier, diarrhea, control, or non-CDI (which combines all other three groups) (13).

Fungi are a significant proportion of the gut microbiota, but their roles in the development of CDI is understudied. Very few mycobiota studies have so far been limited to the comparison between CDI patients and healthy control (14) or non-CDI diarrhea patients (15–17), with relatively small sample sizes. For example, in a study of 24 inpatients with diarrhea (12 of whom had CDI) reported that only one genus, Penicillium, was found predominant in CDI (17). Our previous study revealed that Cladosporium and Aspergillus were enriched in carrier and control with respect to CDI (18). Meanwhile, it is uncertain whether the observed gut mycobiota changes could be used for distinguishing CDI from non-CDI and/or diarrhea. Our previous research revealed that specific serum immune factors distinguish CDI from carrier (5). However, the feasibility of using mycobiota signatures to distinguish CDI from non-CDI inpatients or non-CDI diarrhea has not been studied.

Here, we designed a study in a well characterized cohort of 149 patients that consists of both CDI and non-CDI patients. We aimed to analyze gut mycobiota composition from individuals with CDI and non-CDI or diarrhea using internal transcribed spacer 2 (ITS2) sequencing. Our study provides new evidence for mycobiota alterations among large group of well characterized inpatients, which could help expand the understanding of the relationship between mycobiota and CDI and direct new diagnostic efforts toward CDI from non-CDI or diarrhea. We also tested whether our candidate immune factors (18) combined with fungal markers could serve as signatures that effectively distinguish CDI patients from other hospitalized patients.

## RESULTS

### Description of study population.

Stool samples were prospectively collected from 149 patients at Beth Israel Deaconess Medical Center containing 58 in the CDI group and 91 in the non-CDI group (including 28 carrier, 31 diarrhea, and 32 control). We found no significant differences in the clinical characteristics of these patients, including sex, age, and race, between these cohorts (*P > *0.05 for each; Table S1). Permutational multivariate analysis of variance (PERMANOVA) indicated that the groups and the clinical characteristics of the participants, including sex, age, and race, had no significant impact on the fungal composition (*P > *0.05; Table S2).

### Sequence characteristics.

After quality filtering steps, a total of 9,279,352 filtered sequences were obtained from all 149 patients, with 62,274 sequences per sample on average (SD = 6,320). The UNITE ITS reference data set was used for determining operational taxonomic units (OTU) at the 97% level. The average coverage of the generated OTUs reached up to 99.9% (Fig. S1), and the rarefaction curves achieved the even stage (Fig. S2A and B), suggesting that the libraries were sufficiently large to capture most of the fungal diversity in the samples. The number of sequences assignable to known taxa in the database gradually decreased from phylum to species. Overall, we detected of 6 phyla, 29 classes, 77 orders, 172 families, 290 genera, 435 species, and 776 OTUs in the stool samples. A Venn diagram depicts the common and unique between two cohorts (Fig. S2D). Using OTU counts, a total of 194 OTUs, accounting for 25.00% of the total abundance, were shared between CDI and non-CDI. There were 130 and 452 OTUs exclusive to the CDI and non-CDI groups, respectively (Fig. S2C). There were 126 common fungal OTUs shared in the CDI and diarrhea groups, and 198 and 99 unique OTUs were identified in the CDI and diarrhea groups, respectively. These data suggested that the general properties of fungal mycobiota differed in this study group.

### Ecological features of the fecal fungal flora.

**FIG 1:**
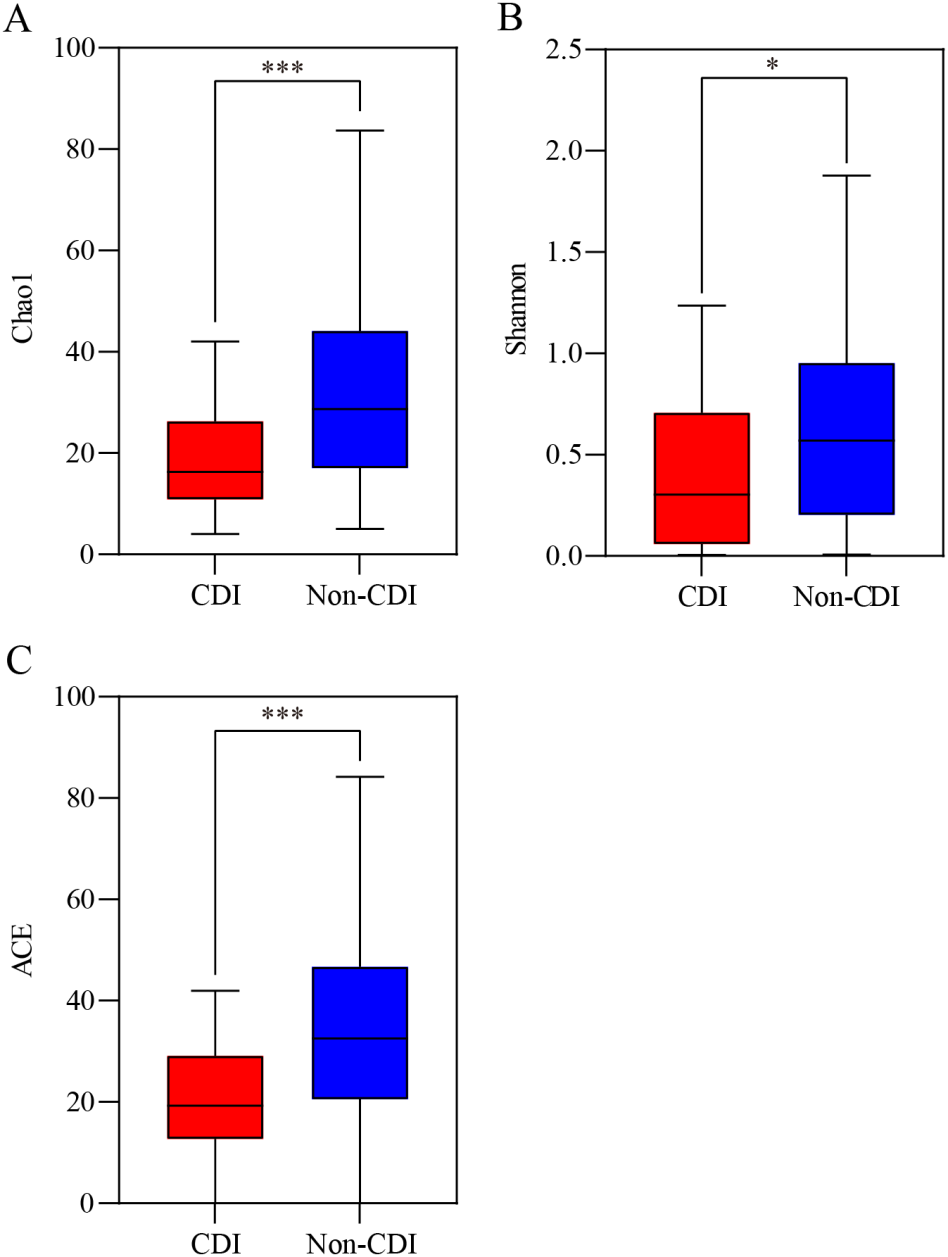
α-Diversity of fungal taxa at operational taxonomic units (OTU) level between C. difficile infection (CDI) and non-CDI. (A) Chao1. (B) Shannon diversity. (C) Abundance-based coverage estimator (ACE) indexes. *, *P < *0.05; ***, *P < *0.001.

We characterized the ecological features of the fecal fungal flora using a variety of α-diversity indices at the OTU level. The indices of Chao1, Shannon, abundance-based coverage estimator [ACE], and coverage were calculated to analyze the richness and diversity of all the samples. The fungal α-diversity parameters, such as Chao1, Shannon, and ACE (Fig. 1), in the CDI group were lower than those in the non-CDI group (*P < *0.05), suggesting that CDI had lower fungal diversity and richness than non-CDI. Not all measured α-diversity indices were significantly different between the CDI and diarrhea groups (Fig. S3), indicating similar levels of diversity and richness of the fungal communities in these two groups.

**FIG 2:**
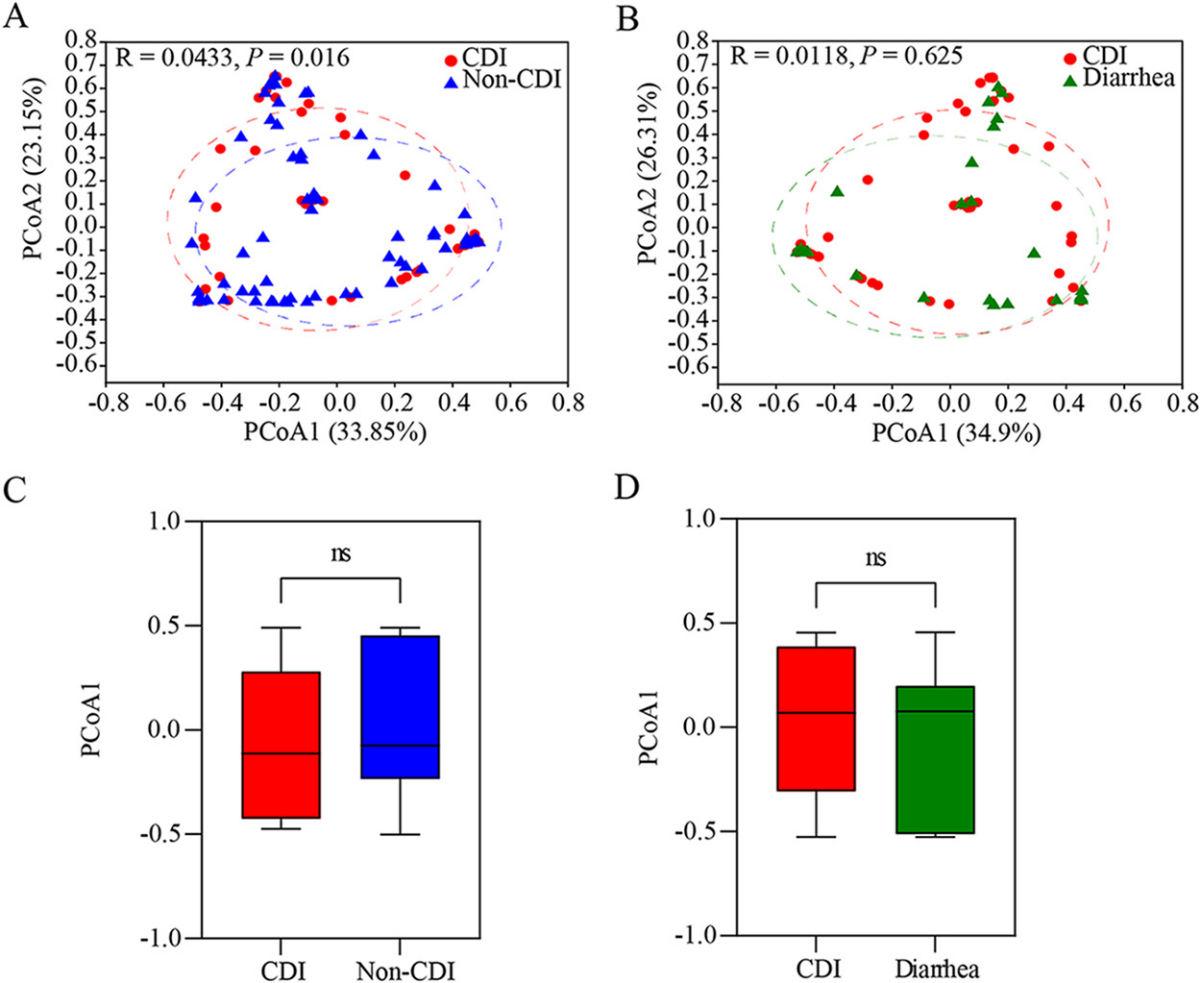
β-Diversity of fungal taxa at operational taxonomic units (OTU) level between *C. difficile* infection (CDI) and non-CDI or diarrhea. (A, B) Principle coordinate analysis (PCoA) based on the Bray-Curtis dissimilarity matrix between CDI and non-CDI (A) or between CDI and diarrhea (B). The ellipses represent 95% confidence regions for each group. (C, D) The PCoA1 within two cohorts were compared. ns, *P > *0.05.

We performed principal coordinate analysis (PCoA) at the OTU level to analyze the β-diversity in fungal composition. PCoA based on Bray-Curtis dissimilarity demonstrated significant differences in gut taxonomic composition between the CDI and non-CDI cohorts (*R*_ANOSIM_ = 0.0433; *P = *0.016; Fig. 2A). The highest PCoA variations in the fungal were 33.85% (PCoA1) and 23.15% (PCoA2), representing a strong separation of different samples between CDI and non-CDI. There was not a clear differentiation between the CDI and diarrhea groups in the PCoA and ANOSIM results (*R*_ANOSIM_ = 0.0118; *P = *0.625; Fig. 2B). These data suggested that gut mycobiota of diarrhea patients was more consistent, while the fungal structure and composition of CDI were significantly different than those of non-CDI.

### Taxonomic distribution and differential abundance analysis.

We further analyzed the taxonomic abundance of fecal samples at the phylum and genus levels. Ascomycota and Basidiomycota were the dominant fungal phyla, with Ascomycota surpassing 90% of the sequences in all groups (Fig. S4A). Moreover, Candida, Saccharomyces, and Nakaseomyces were the dominant fungal genera in all cohorts (Fig. S4B).

**FIG 3:**
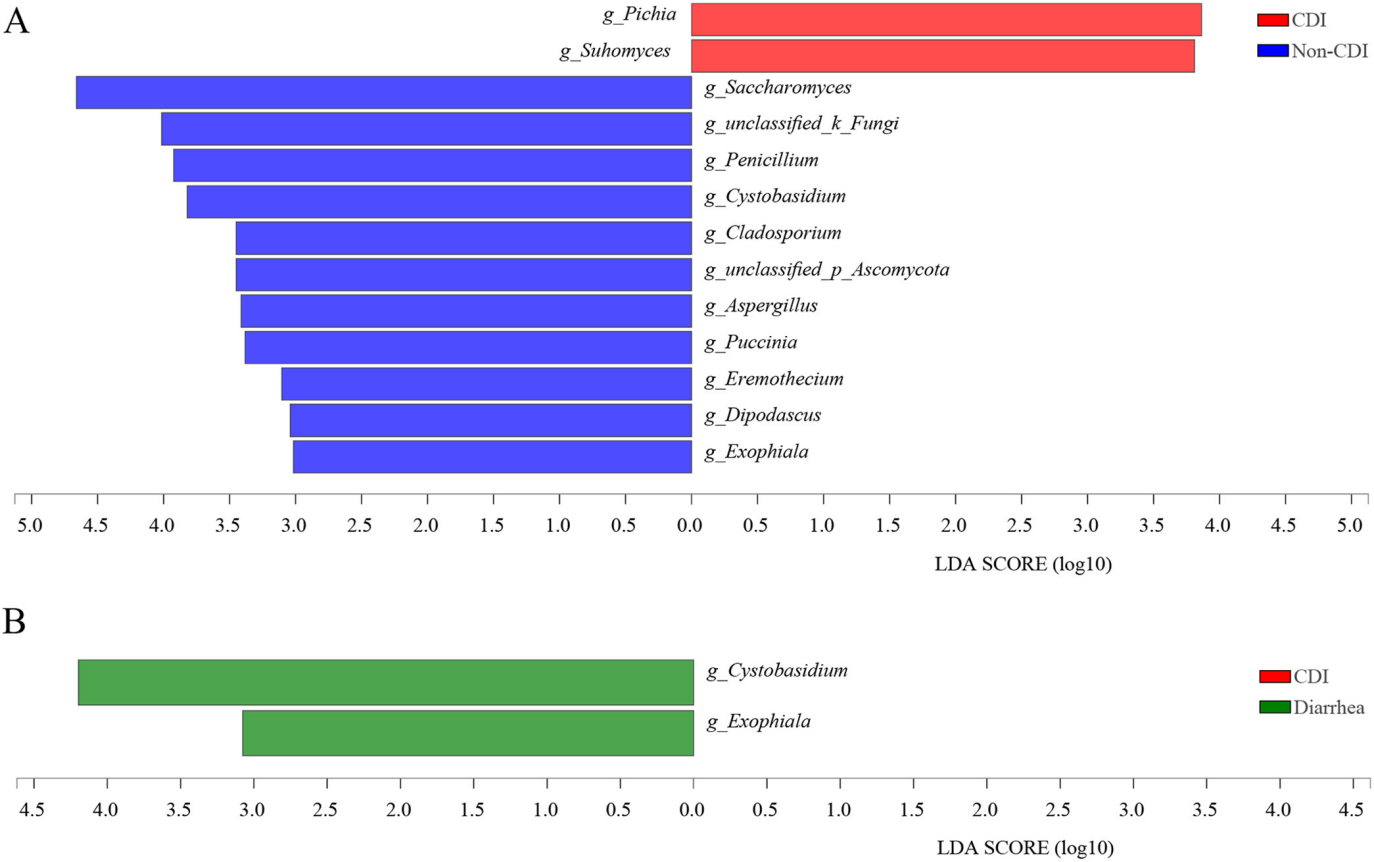
Linear discriminant analysis (LDA) of effect size (LEfSe) of fungal taxa at genus levels in fecal samples from *C. difficile* infection (CDI) (red), non-CDI (blue), and diarrhea (green). LEfSe is an algorithm to identify high-dimensional biomarkers, and only species meeting an LDA significant threshold of >3.0 and *P < *0.05 were shown. “Unclassified” refers to fungal taxa not assigned by the UNITE database.

The predominant fungi were largely consistent at phyla and genera levels, but different relative abundances could be observed. To further identify differentially abundant taxa, we performed linear discriminant analysis of effect size (LEfSe) to compare relative abundance genera between CDI and non-CDI/diarrhea subjects. The phylum of Ascomycota was significantly higher in the CDI group than in the non-CDI group, while the phylum of Basidiomycota was significantly lower in the CDI group than in the non-CDI group (Fig. S5). Meanwhile, discrepancies were detected between the CDI and non-CDI groups, with a higher abundance of Pichia and Suhomyces in the CDI group and a higher abundance of 11 genera (Saccharomyces, Penicillium, Aspergillus, Cystobasidium, Cladosporium, and so on) observed in the non-CDI group at the genus level (LDA >3.0; *P < *0.05; Fig. 3A). Comparing relative abundance between CDI and diarrhea subjects, no significant change was observed at the phylum and genus levels (LDA >3.0, *P < *0.05; Fig. 3B), except the genera Cystobasidium and Exophiala, which were significant lower in the CDI group than in the diarrhea group (*P < *0.05).

**FIG 4:**
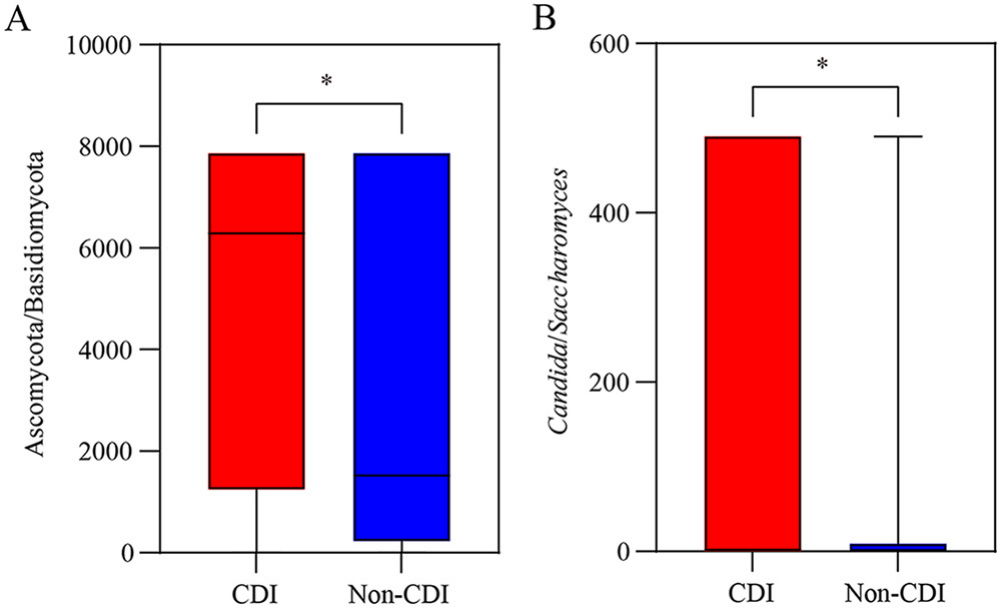
The Ascomycota to Basidiomycota ratio (A) and *Candida* to *Saccharomyces* ratio (B) of gut mycobiota from *C. difficile* infection (CDI) and non-CDI. The Data are presented as median and 95% confidence interval, and the *P* values were based on *t* test analysis. *, *P < *0.05.

**FIG 5:**
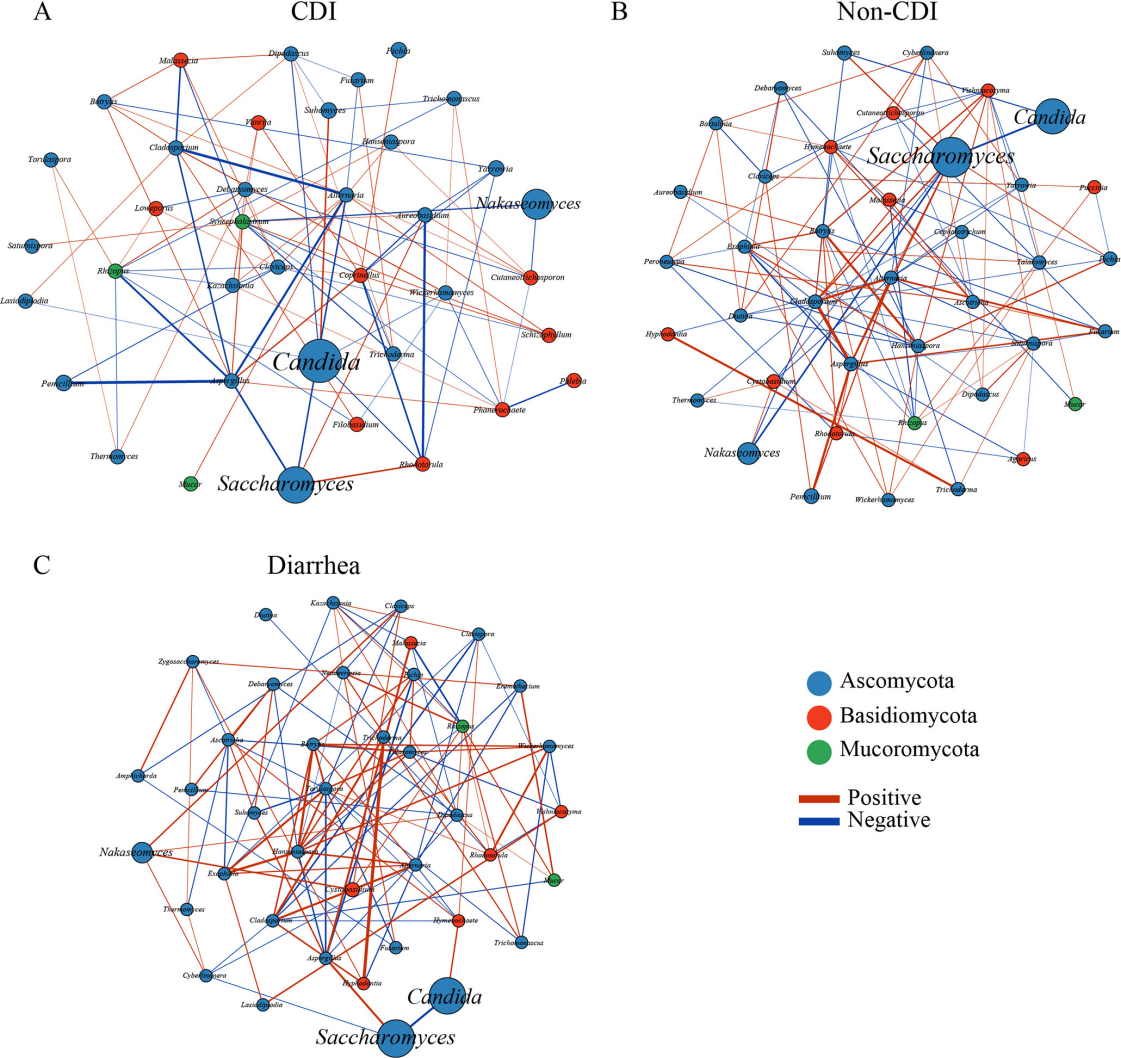
Fungal correlation networks from *C. difficile* infection (CDI) (A), non-CDI (B), and diarrhea (C). Network analysis showed interactions of 40 richest genera. The genera are represented as nodes, and abundance is represented by node size. Node color corresponds to phylum taxonomic classification. Edges between nodes represent fungal correlations between the nodes, with edge color indicating positive (green) and negative (red) correlations, respectively. The absolute value of the correlation coefficient is represented by the edge thickness, and only absolute correlation coefficients > 0.1 with *P < *0.05 are presented.

Spearman’s correlation analysis revealed that the phylum Ascomycota was negatively correlated with the phylum Basidiomycota (*r* ≤ −0.714; *P < *0.05; Fig. S6). Consequently, the Ascomycota-to-Basidiomycota ratio was greater in the CDI group than in the non-CDI group (*P < *0.05; Fig. 4A), while no significant difference was identified between the CDI and diarrhea groups (*P > *0.05; Fig. S7). Although Candida was not associated with CDI in our study, we found that Candida was negatively correlated with the Saccharomyces (*r* ≤ −0.387; *P < *0.05; Fig. 5). The Candida-to-Saccharomyces ratio was significantly higher in the CDI group than in the non-CDI group (*P < *0.05; Fig. 4B), while no significant difference was identified between the CDI group and the diarrhea group (*P > *0.05; Fig. S7). These data indicate that the ratios of Ascomycota to Basidiomycota and Candida to Saccharomyces, as a fungal dysbiosis index, may be useful to differentiate CDI from non-CDI, in addition to differentiating CDI from asymptomatic carriers (18).

### Fungal correlation networks.

Network analysis was performed to understand associations among genera in different niches. In this study, a unique structure of the fungal correlation network was found in the CDI group. The overall fungal correlations appeared much weaker in the CDI group than in the non-CDI group (Fig. 5). In addition, some fungal correlations disappeared in the CDI group, in contrast to the non-CDI group.

### Correlation analysis between mycobiota features and serum immune factors.

**FIG 6:**
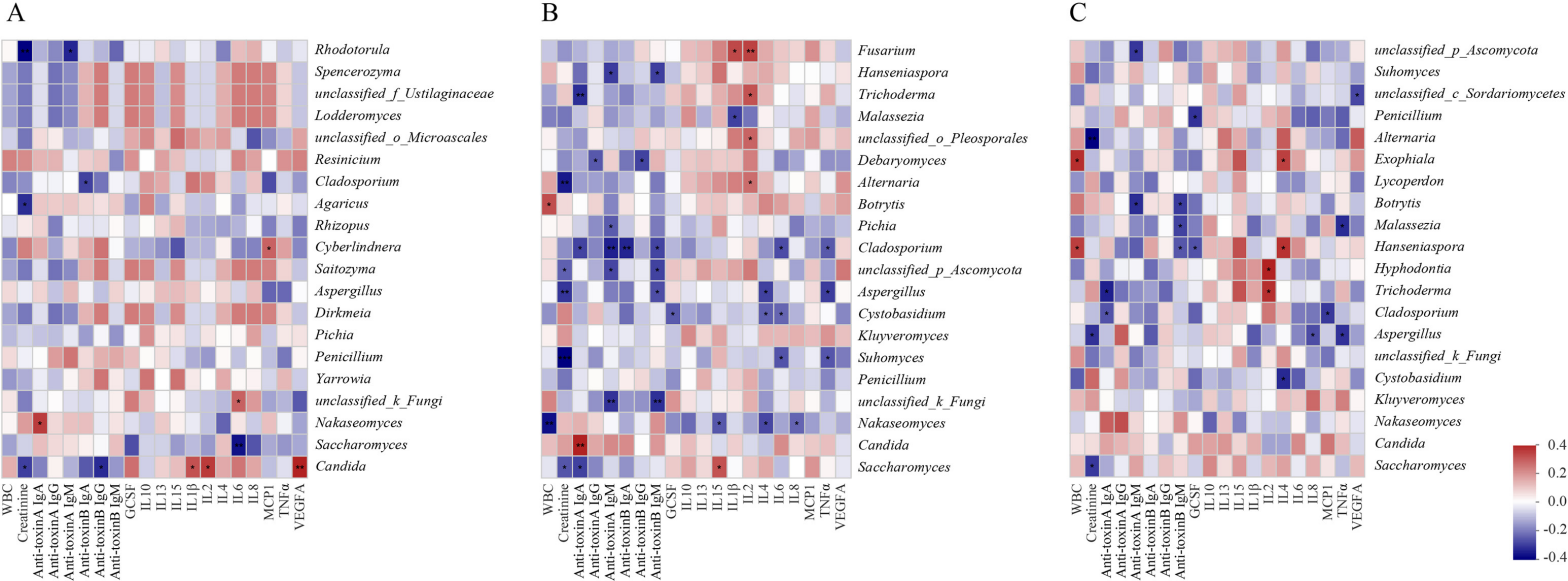
Spearman correlations between fungal communities and serum immune factors from *C. difficile* infection (CDI) (A), non-CDI (B), and diarrhea (C). The rows display the fungal taxa at the genus level and, the columns represent the immune factors. Red, positive correlations; blue, negative correlations. The intensity of the color represents the degree of association between fungal communities and serum immune factors. *, *P < *0.05; **, *P < *0.01; ***, *P < *0.001. WBC, white blood cell; GCSF, granulocyte colony-stimulating factor; IL, interleukin; MCP, monocyte chemoattractant protein; TNF, tumor necrosis factor; VEGF, vascular endothelial growth factor.

The correlations between fungal compositions and serum immune factors showed a significantly smaller number of positive and negative correlations in the CDI group compared to the non-CDI and diarrhea groups (Fig. 6). Interestingly, unique correlations between fungal compositions and serum immune factors were observed in each group.

## DISCUSSION

Increasing although limited evidence has been emerging to support the role of mycobiota in CDI. We previously reported a diagnostic model combining specific fungi and serum immune factors with diagnostic potential to distinguish CDI patients from carriers (18). With an expanded clinical cohort in our current study, we focused on the difference of fecal mycobiota between the CDI group and the non-CDI or non-CDI diarrhea group, which have not been extensively evaluated before. We found that inpatients with CDI demonstrated fungal dysbiosis compared with all non-CDI subjects characterized by a lower α-richness. Eleven genera (Saccharomyces, Penicillium, Aspergillus, Cystobasidium, Cladosporium, and so on) were significantly enriched in non-CDI patients, and Pichia and Suhomyces were enriched in CDI, while two genera abundance of Cystobasidium and Exophiala were higher in patients with diarrhea compared to those with CDI. The ratios of Ascomycota to Basidiomycota or Candida to Saccharomyces could be used as a valuable biomarker to differentiate CDI from non-CDI group. This expanded the potential of this fungal dysbiosis index in clinical diagnosis, as we previously reported that it can differentiate CDI patients from carriers (18). A disease-specific pattern with strong fungal abundance correlations in the non-CDI and diarrhea groups, which were absent in CDI, suggested that these correlations may contribute to the development of CDI. Along with our recent reports on CDI patients versus carriers (18), associations between mycobiome and immune factors could imply that host-mycobiome interactions also exist in the CDI group and the non-CDI or diarrhea group.

The role of the gut mycobiota in CDI is understudied mainly due to the technical challenges of ITS sequencing and the limitations of fungal databases, resulting in many fungal taxa *incertae sedis* (16). The cohort population in this study had a relatively large group of well characterized patients, which enhanced the power of our analysis. Deep sequencing coverage revealed diverse fungal communities existed in both the CDI and non-CDI/diarrhea groups, as samples in all cohorts had a high-quality fungal read per sample. In addition, coverage and rarefaction curves results in this study suggested that the libraries were sufficiently large to capture most of the fungal diversity in the samples.

As expected, the α-diversity indices, in this case Chao1, Shannon, and ACE, were significantly lower in the CDI group than in the non-CDI group. No significant difference between CDI and diarrhea were observed, consistent with previous observational studies (14). β-Diversity showed evident separation between CDI and non-CDI patients. This finding is consistent with our previous study, in which significant differences were identified in α- and β-diversity between the CDI and carrier cohorts (*P < *0.05) (18). A previous study also found that the fungal communities in CDI cohorts were separated from healthy controls at the OTU level based on Bray-Curtis dissimilarities (*P = *0.003) (14). A mixed pattern was found between the CDI and diarrhea groups in this study, while a previous study showed that significant differences in fungal community composition were found between the CDI and diarrhea cohorts (*P = *0.038) based on PCoA analysis (15). A prior study indicated that CDI patients could be separated from diarrhea and healthy control patients, whereas diarrhea patients could not be distinguished from healthy control (principal component analysis [PCA] and PCoA) (19). Therefore, the result of β-diversity between the CDI and diarrhea groups remains controversial. The observed differences could be partly attributed to sample size, sequencing methods, sequencing depths, individual variations, and some other factors.

In our current study, the fungal mycobiota in the CDI and non-CDI (or diarrhea) groups was dominated by the phyla Ascomycota and Basidiomycota and the genera Candida and Saccharomyces. The phylum Ascomycota was significantly higher in CDI than in non-CDI, while the phylum Basidiomycota and the genus Saccharomyces were significantly lower in CDI than in non-CDI. Meanwhile, the phyla Ascomycota and Basidiomycota and the genera Candida and Saccharomyces were strongly negatively correlated with each other in this study; thus, the ratios of Ascomycota to Basidiomycota or Candida to Saccharomyces were higher in the CDI group than in the non-CDI group (*P < *0.05). In a recent study, we, too, found that the ratio of Ascomycota to Basidiomycota was significantly higher in CDI than in the carrier or control groups (*P < *0.05) (18). A previous study also revealed that Ascomycota was expanded in CDI in comparison to healthy controls (14) or non-CDI diarrhea patients (16) at the phylum level. Several clinical studies have examined the effectiveness of Saccharomyces boulardii CNCM I-745 as an intervention to prevent the development of CDI, as well as to reduce recurrent CDI (20–23). Overrepresentation of Candida and Candida albicans were frequently observed in CDI (14, 24, 25). In this study, Candida was the most abundant genera in CDI, but it was not significantly different between the CDI and non-CDI cohorts. This might be due to the great heterogeneity in the Candida genus. In addition, another reason could be the challenge in distinguishing fungi at the species level by using the current ITS2 sequence methods (18). Previous findings revealed that Pichia is a group with some species reclassified as Candida (26). This maybe the reason why Pichia were higher in the CDI group than in the non-CDI group, while Candida were not in this study. These ratios used to define the fungal dysbiosis suggest that an increased fungal Ascomycota-to-Basidiomycota ratio or Candida-to-Saccharomyces ratio and altered fungal diversity may be associated with the pathogenic features of CDI. Meanwhile, these ratios could be potential biomarkers to differentiate CDI from non-CDI.

In the LEfSe test, the fungal genera abundance of Pichia and Suhomyces were higher in the CDI group, while Saccharomyces, Penicillium, Aspergillus, Cystobasidium, and Cladosporium were higher in the non-CDI group. These findings are inconsistent with a previous small study that Byssochlamys and Helotiales were significantly enriched within the CDI patients (15). LEfSe revealed that 17 fungi were significantly different between the CDI and control groups at the species level, and among these species, only C. albicans was significantly enriched in CDI (*P = *0.008), whereas 16 other species were enriched in the control group, indicated dysbiosis of the gut fungi in CDI cohort (14). Previous study has demonstrated that *Penicillium* genus were more frequently associated with CDI compared to controls among inpatients with diarrhea (17). An animal study has also reported that Penicillium was a predominant fungal element in the hamster gut microbiome when clindamycin was used to induce CDI (27). The higher abundance ratios of the Penicillium genus in CDI therefore suggest their possible contribution toward CDI pathogenesis and warrants further investigations.

In our study, the fungal genera Aspergillus and Cladosporium significantly decreased in the CDI group compared to the non-CDI group. This is consistent with our previous findings in CDI patients versus carriers (18). Another study also reported that Aspergillus was found to be enriched in healthy individuals compared to CDI (14). Aspergillus is a major component of the fungal community in hamsters, while treated C. difficile with clindamycin and exogenous monoclonal antibody, the ultimate community was similar but altered by decreased numbers of Aspergillus (27). So far, there is no published investigation on the relationship between Cladosporium and CDI. The decrease of Aspergillus and Cladosporium in CDI identified in this study and our previous one (18) highlights the potential beneficial role of these fungi in the gut, suggesting a therapeutic approach for CDI. Numerous studies have revealed that cytokine (including interleukin-1β [IL-1β], IL-10, tumor necrosis factor α [TNF-α], and so on) increased as resting conidia of A. fumigatus germinated into hyphae or the phagocytic activity of macrophages (28, 29). This is why several cytokines were negative with Aspergillus in this study. Aspergillus has also been reported to be involved in several severe inflammatory conditions, including Crohn’s disease (30). Taken together, it is plausible that Aspergillus contribute to effect of immune systems, although the effect may not be disease specific.

In this study, LEfSe revealed that the genus Cystobasidium was prominent in diarrhea compared with CDI, and Cystobasidium were found to be decreased in CDI patients compared to non-CDI patients. We also found that Cystobasidium exhibited complicated negative interactions with IL-4. To our knowledge, no studies were done on the role of and relationship between genera Cystobasidium and diarrhea with or without CDI. This is the first study to describe such an association in human subjects, and the Cystobasidium may be a potential biomarker for differentiation of CDI from non-CDI diarrhea. The role of Cystobasidium and IL-4 in the pathogenesis of CDI and diarrhea warrant further studies in CDI patients and animal models.

The analysis of the mycobiota of CDI patients and non-CDI or diarrhea patients clearly showed distinctive fungal interactions among stool mycobiota, indicating that some fungal relationships may be disrupted and result in dysbiosis in the CDI process. These correlation fungi might be essential fungi that also have essential roles in the gut. Thus, we found some co-occurring fungi correlations disappeared, comparing CDI with non-CDI or diarrhea, suggesting that these correlations may contribute to prevent CDI development. The correlation analysis improved our understanding the partnership in the inpatient stool samples. Therefore, future studies need to focus on exploring the physiology of these disappearances of some co-occurring fungi correlations. Such disappearance correlations within the fungal network may provide a potential objective for novel CDI therapy.

In this study, a weak fungal correlation was observed in CDI group than in the non-CDI and diarrhea groups, suggesting that these correlations may contribute to the development of CDI. The absence of these fungi and immune factor correlations may mediate CDI susceptibility (18). However, the complex directionality of these interactions between fungi and immune factors in this research still need to be explored in further studies.

Mycobiota and microbiota are habitats of the human gut and occupy the same ecological niche. They develop intricate interactions such as obtaining nutrients required for proliferation and colonization from each other (31). The homeostasis of the mycobiota and microbiota ecological network protects the host from dysbiosis-related disease (32). Previous studies have revealed that specific fungi could alter the bacterial community (33). Bacterial microbiota dysbiosis, extensive tissue damage, and the presence of an inflammatory environment could cause gut fungal overgrowth (30). Fungal dysbiosis was associated with reduced efficacy of fecal microbiota transplantation in CDI (14). Mycobiota and microbiota inevitably interact with each other. Their interactions in CDI patients and other diseases will be the highlights of future research.

### Conclusion.

The data reported here extend our understanding of mycobiota composition in CDI patients and highlight the need for additional research to further understand its potential impacts on disease diagnosis and pathogenesis. Immune factors with or without the fungi from this study provide novel insights that may be valuable for raising the possibility of using these biomarkers in the stratification of CDI patients from other patient groups.

## MATERIALS AND METHODS

### Patient cohorts.

The background and design of these cohorts have been detailed in our previous studies (5, 6, 13). All individuals were age 18 years and older. CDI patients had positive clinical stool NAAT results with diarrhea and were treated for CDI. Carrier patients included those admitted for at least 72 h, those who had received at least one dose of an antibiotic within the past 7 days, and those without diarrhea in the 48 h before fecal sample collection but had positive NAAT results and were not treated for CDI. Diarrhea patients included individuals with negative NAAT who had diarrhea (confirmed using the same definition as the CDI cohort) caused by other factors. Control patients were NAAT-negative without diarrhea. Serum samples were collected as discards within 24 h of fecal sample collection. The last three groups were combined as the non-CDI group.

This study was approved by the institutional review board (IRB) of Beth Israel Deaconess Medical Center (IRB protocols 2016P000026 and 2016P000054). All human subjects provided informed consent for participation in the study and collection and analysis of data.

### Fungal ITS2 sequencing and bioinformatics analysis.

The details of the fungal sequencing and bioinformatics analysis are available in our previous study (18). The isolation of fecal DNA was conducted by using the QIAamp DNA stool minikit (Qiagen, Hilden, Germany) in accordance with Qiagen standard protocol. The fecal DNA for fungal sequencing was amplified based on ITS2 region using the PrimeSTAR HS DNA polymerase kit (TaKaRa Shuzo, Kyoto, Japan) with primers (forward primer: 5′-GCATCGATGAAGAACGCAGC-3′ and reverse primer: 5′-TCCTCCGCTTATTGATATGC-3′). Illumina Hiseq 2500 platform was used for ITS2 sequencing. Sequencing library preparation and data processing were developed by BGI Genomics (Shenzhen, Guangdong, China) (14).

### Data analysis.

α-Richness (Chao1 and ACE) and diversity (Shannon) were compared in accordance with previous study (34). Permutational multivariate analysis of variance was determined based on the Adonis and the Bray-Curtis with the default 999 permutations (35). Differences in the relative abundance of the detected mycobiota were determined by LEfSe, with LDA > 3.0 and *P < *0.05 considered significant (36). The Ascomycota-to-Basidiomycota ratio (or Candida-to-Saccharomyces ratio) was calculated based on *t* test results. Fungal correlation network was constructed using Sparse Correlations for Compositional (SparCC) (37). The absolute value of sparse correlation |*r*| > 0.1 and *P < *0.05 were used for selecting correlated genus pairs. Correlations between the mycobiota and serum immune markers were determined by using Spearman correlations analysis with the default of *P < *0.05 and to be detected in ≥15% of all samples in each group. In this study, SparCC analysis was based on python, while all other data analysis was performed using R.

### Data availability.

The sequencing data that support the findings of this study have been deposited in the National Center for Biotechnology Information Sequence Read Archive under BioProject ID PRJNA764417.
