# Supplementary material for: Analysis of Intestinal Mycobiota of Patients with Clostridioides difficile Infection among a Prospective Inpatient Cohort
Source: Microbiol Spectr. 2022 Jul 14;10(4):e01362-22. doi: 10.1128/spectrum.01362-22 (PMC9430669; doi:10.1128/spectrum.01362-22)
Supplement: Supplemental file 1 — Supplemental material. Download spectrum.01362-22-s0001.pdf, PDF file, 0.9 MB [file spectrum.01362-22-s0001.pdf]

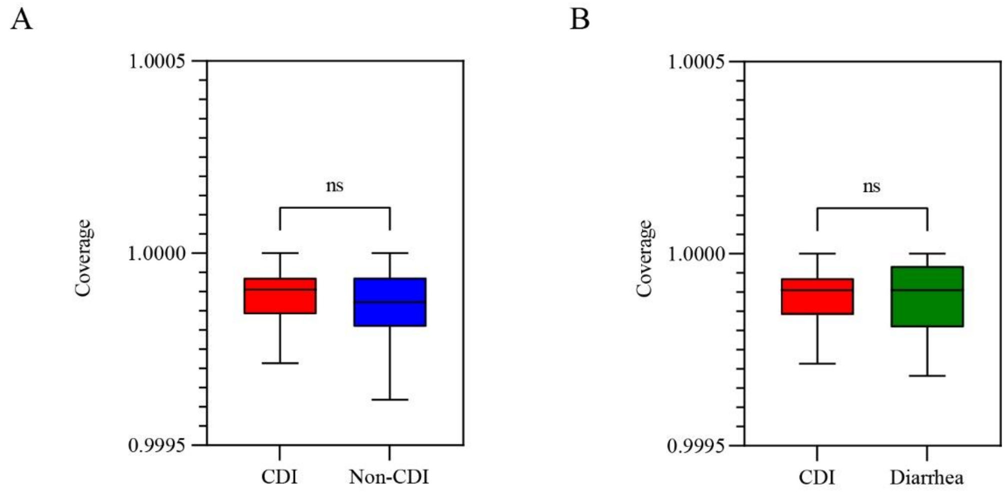

Fig S1 Coverage of fungal taxa at OTU level between CDI and Non-CDI (A) or Diarrhea (B). ns $P > 0.05$.

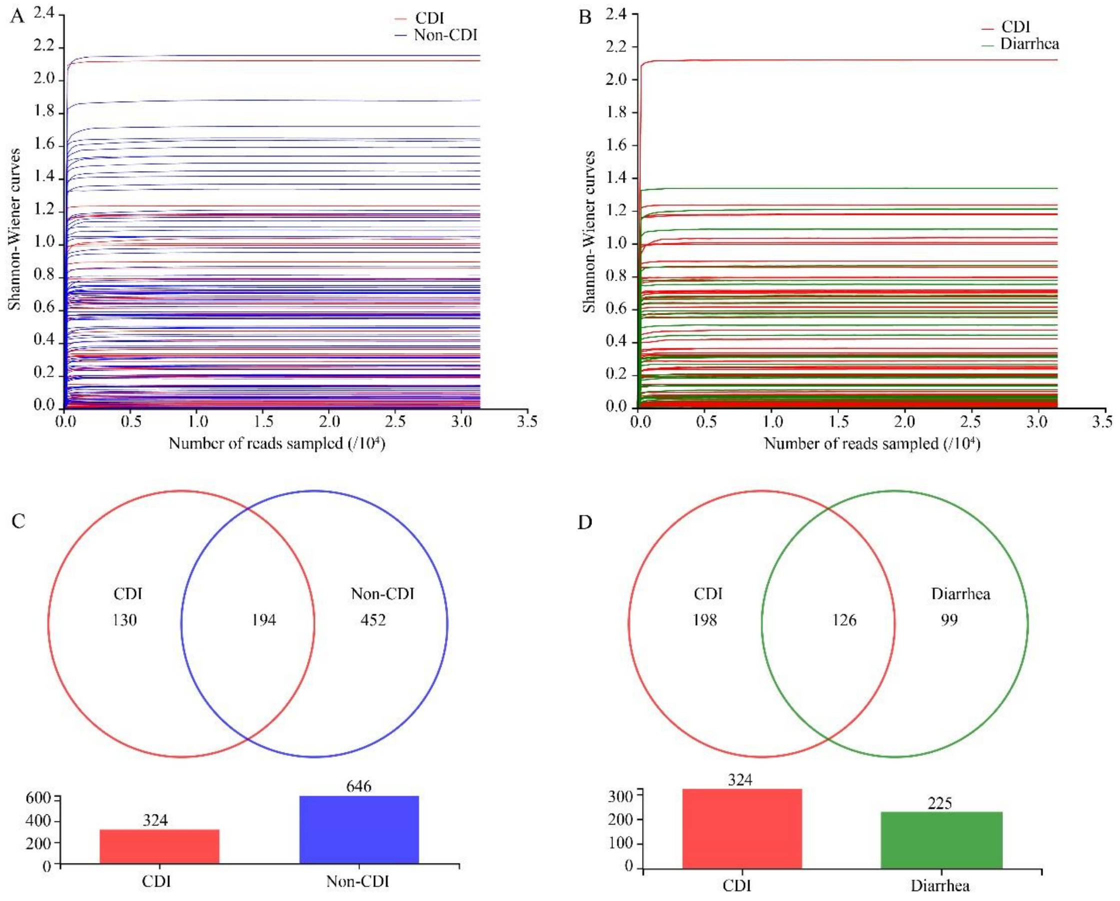

Fig S2 Shannon-Wiener curves and Venn diagram of fungal taxa in fecal samples from CDI, Non-CDI and Diarrhea. (A-B) Shannon-Wiener curves were all calculated at a 0.03 dissimilarity level. Shannon-Wiener curves were collated from each sample's Shannon diversity index. The graphic shows the estimated diversity plotted against the number of sequences per sample. Each line represents one sample. The plateau in each estimated diversity curve indicates the minimum number of sequences to capture diversity. (C-D) A Venn diagram showing shared and unique OTUs at a 0.03 dissimilarity level.

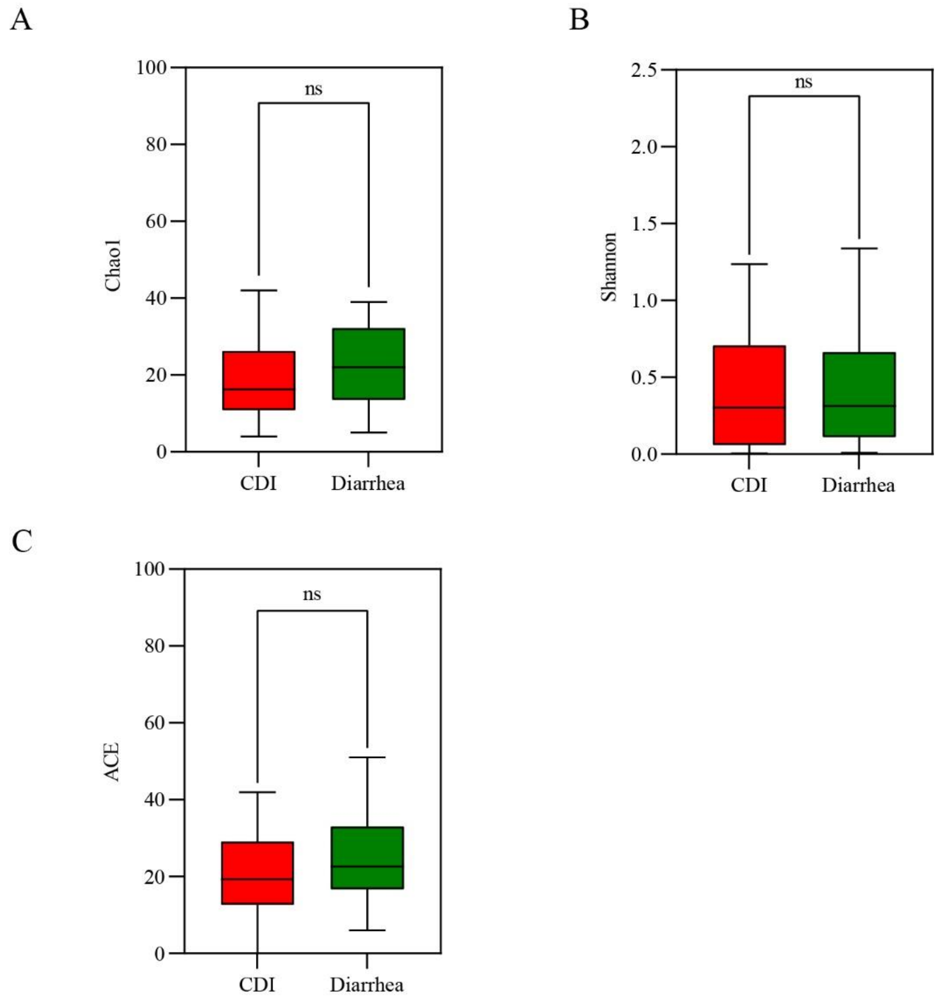

Fig S3 Alpha diversity of fungal taxa at OTU level between CDI and Diarrhea. The analysis was based on: (A) Chao1, (B) Shannon diversity, (C) ACE indexes. ns $P >$ 0.05.

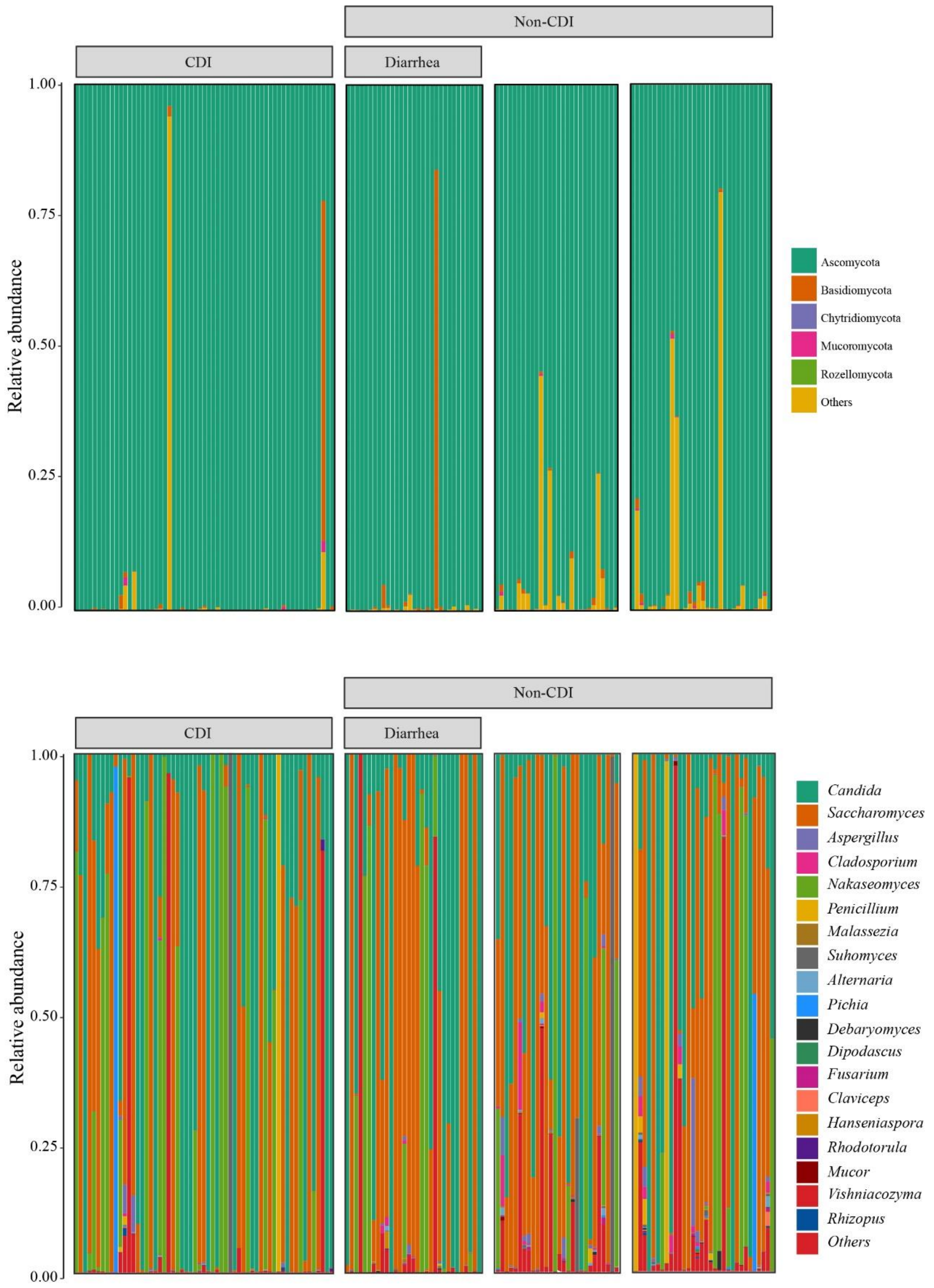

Fig S4 Genus-level and phylum-level taxonomic profiles of the gut mycobiota. Only genera or phylum with ≥ 1% abundance in at least one sample were depicted. Otherwise, they were included in the category "others".

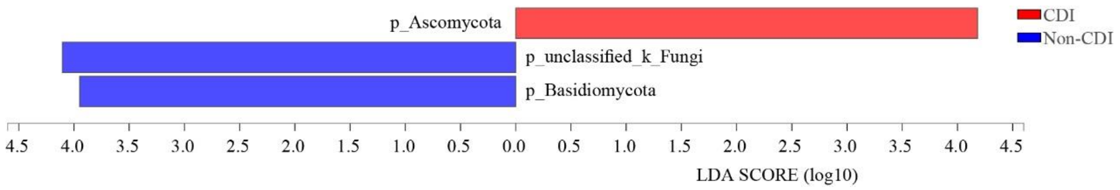

Fig S5 Linear discriminant analysis (LDA) effect size (LEfSe) test of fungal taxa at phylum levels in fecal samples from CDI (Red) and Non-CDI (Blue). Linear discriminant analysis (LDA) effect size (LEfSe) is a tool to identify biomarkers from high dimensional data of 2 or more groups using. Only species meeting an LDA significant threshold > 3.0 and $P < 0.05$ were shown. The unclassified refer to fungal taxa not assigned by the unite database.

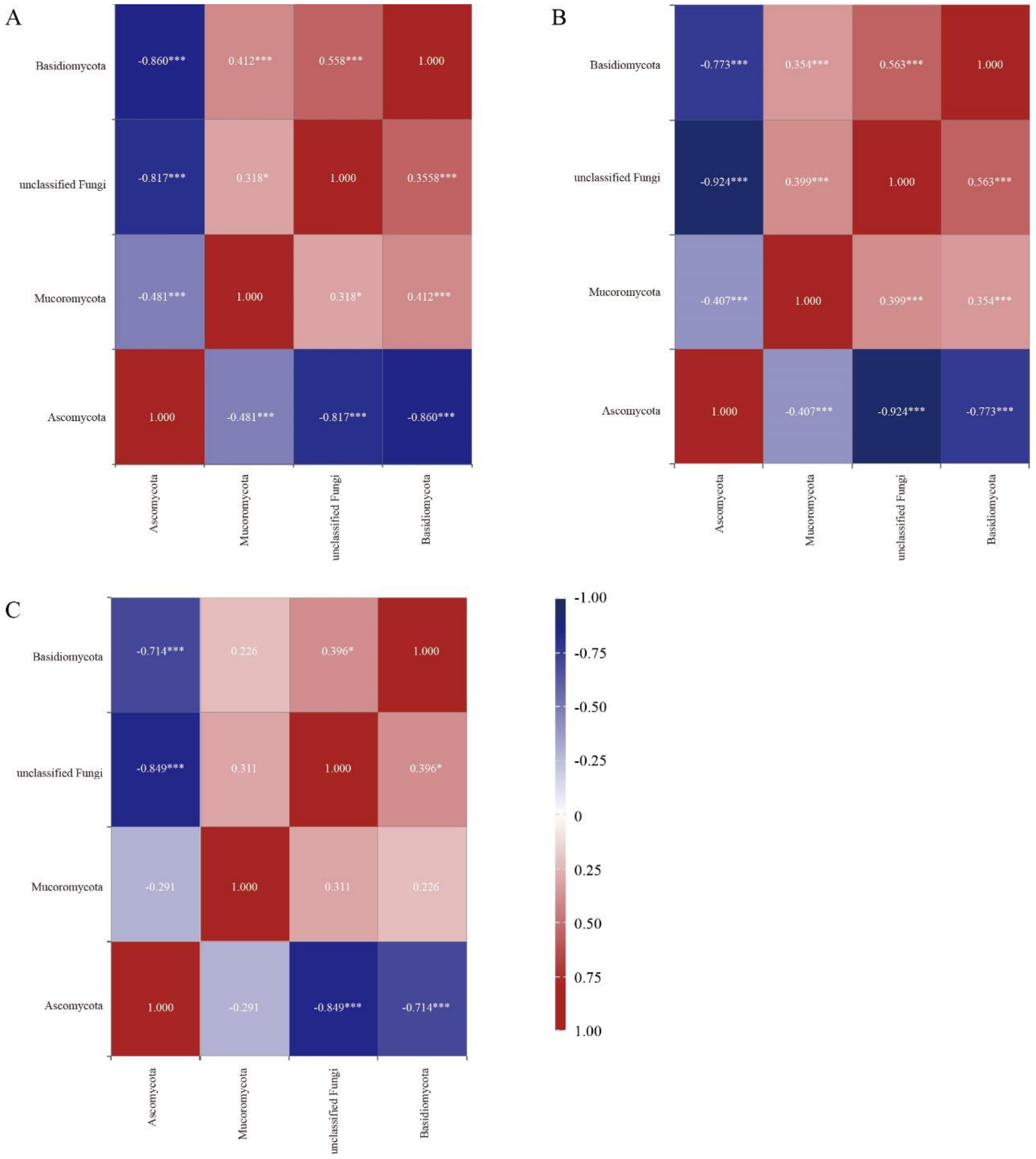

Fig S6 Correlation of fungal taxa at phylum levels at the CDI (A), Non-CDI (B) and Diarrhea (C) groups. Red squares indicate positive correlations, and blue squares indicate inverse correlations. The sign of the correlation was determined using Spearman's method. *$P < 0.05$, **$P < 0.01$, ***$P < 0.001$.

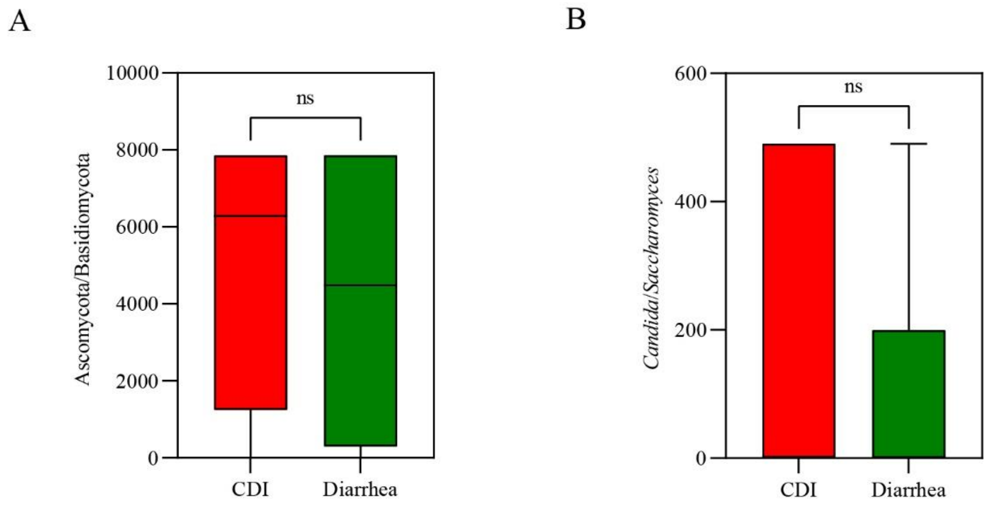

Fig S7 The Ascomycota to Basidiomycota ratio (A) and *Candida* to *Saccharomyces* ratio (B) of gut mycobiota from CDI and Diarrhea. Data are presented as median and 95% CI with *P* values based on T test. ns $P > 0.05$.

Table S1 Demographic characteristics of the enrolled subjects

Characteristics	CDI (n = 58)	Non-CDI (n = 91)	Diarrhea (n = 31)	*P* value (CDI vs Non-CDI)	*P* value (CDI vs Diarrhea)
**Sex**				0.395#	0.823#
Female	27 (46.55%)	35 (38.46%)	13 (41.94%)		
Male	31 (53.45%)	56 (61.54%)	18 (58.06%)		
**Age, Avg ± SD**	65.38 ± 15.30	60.02 ± 15.15	59.71 ± 12.42	0.060*	0.154*
**Race**				0.449#	0.215#
White	45 (77.59%)	65 (71.43%)	20 (64.52%)		
Others/unknown	13 (22.41%)	26 (28.57%)	11 (35.48%)		

* T test, # Chi square test.

Table S2 PERMANOVA in mycobiota compositions

Characteristics	CDI vs Non-CDI			CDI vs Diarrhea		
	F	R^2	*P* value	F	R^2	*P* value
Cohorts	1.852	0.012	0.114	0.645	0.007	0.611
Sex	0.753	0.005	0.545	0.598	0.007	0.647
Age	2.016	0.014	0.085	1.116	0.013	0.325
Race	1.098	0.039	0.327	0.701	0.041	0.843

NOTE. Race: White and others. Here F represents the F-statistic: a larger F value indicates that the between-group variation is greater than within-group variation. R^2 represents the variation explained by the model. *P* represents the *P* value calculated from permutation.
